# Quality control of radiomic features using 3D-printed CT phantoms

**DOI:** 10.1117/1.JMI.8.3.033505

**Published:** 2021-06-29

**Authors:** Usman Mahmood, Aditya Apte, Christopher Kanan, David D. B. Bates, Giuseppe Corrias, Lorenzo Manneli, Jung Hun Oh, Yusuf Emre Erdi, John Nguyen, Joseph O. Deasy, Amita Shukla-Dave

**Affiliations:** a Memorial Sloan Kettering Cancer Center, Department of Medical Physics, New York, United States; b Rochester Institute of Technology, Department of Imaging Science, Rochester, New York, United States; c Memorial Sloan Kettering Cancer Center, Department of Radiology, New York, United States; d IRCCS SDN, Naples, Italy; e The Cooper Union, New York, United States

**Keywords:** additive manufacturing, computed tomography, radiomics, quantitative imaging

## Abstract

**Purpose**: The lack of standardization in quantitative radiomic measures of tumors seen on computed tomography (CT) scans is generally recognized as an unresolved issue. To develop reliable clinical applications, radiomics must be robust across different CT scan modes, protocols, software, and systems. We demonstrate how custom-designed phantoms, imprinted with human-derived patterns, can provide a straightforward approach to validating longitudinally stable radiomic signature values in a clinical setting.

**Approach**: Described herein is a prototype process to design an anatomically informed 3D-printed radiomic phantom. We used a multimaterial, ultra-high-resolution 3D printer with voxel printing capabilities. Multiple tissue regions of interest (ROIs), from four pancreas tumors, one lung tumor, and a liver background, were extracted from digital imaging and communication in medicine (DICOM) CT exam files and were merged together to develop a multipurpose, circular radiomic phantom (18 cm diameter and 4 cm width). The phantom was scanned 30 times using standard clinical CT protocols to test repeatability. Features that have been found to be prognostic for various diseases were then investigated for their repeatability and reproducibility across different CT scan modes.

**Results**: The structural similarity index between the segment used from the patients’ DICOM image and the phantom CT scan was 0.71. The coefficient variation for all assessed radiomic features was <1.0% across 30 repeat scans of the phantom. The percent deviation (pDV) from the baseline value, which was the mean feature value determined from repeat scans, increased with the application of the lung convolution kernel, changes to the voxel size, and increases in the image noise. Gray level co-occurrence features, contrast, dissimilarity, and entropy were particularly affected by different scan modes, presenting with pDV>±15%.

**Conclusions**: Previously discovered prognostic and popular radiomic features are variable in practice and need to be interpreted with caution or excluded from clinical implementation. Voxel-based 3D printing can reproduce tissue morphology seen on CT exams. We believe that this is a flexible, yet practical, way to design custom phantoms to validate and compare radiomic metrics longitudinally, over time, and across systems.

## 1 Introduction

There is a growing body of literature about the role of quantitative radiomics (QR) metrics as cancer imaging biomarkers for predicting lesion malignancy and the efficacy of treatments.^1^^–^^3^ Although promising, a general lack of standardization and inconsistent performance of QR metrics across different computed tomography (CT) scan modes is well established.^4^^–^^10^ A solution to improve standardization and quality control (QC) of the QR pipeline should include phantoms that can filter unreliable metrics.^11^^–^^13^

Historically, CT equipment operators have used QC phantoms to monitor the imaging performance of clinical scanners.^14^ However, CT QC phantoms are engineered with homogeneous materials that lack the texture or shapes of tumors seen on CT exams. There is evidence that a textured QC phantom may reveal problems across the imaging pipeline that were not apparent with the routinely used^15^ homogeneous QC phantom.^15^ Another study^16^ showed that the local noise and resolution properties of a lesion depend on the background tissue texture when iterative reconstruction is used to reconstruct the image. As CT scanner hardware and software become more technologically sophisticated, the phantom components will need to take on more realistic properties.

Several phantom types have been proposed to study QR feature variability.^9^^,^^17^^–^^20^ In most literature reports, the phantoms are uniform, consist of patterns that may not be found in patient images, or are shaped in a way that is not characteristic of patient anatomy.^21^ For example, an updated version of the credence cartridge radiomic phantom^17^ consists of six oval cartridges encased in a high-density polystyrene buildup material. The cylindrical shape and encasing are modeled after the size or shape of human anatomy, but the six-round cartridges are placed within a uniform surrounding with each cartridge consisting of a single textured pattern. Evaluating the impact of iterative reconstruction schemes is challenging when the background textures are uniform.^6^^,^^22^ Further, the homogeneous shapes will not be able to evaluate interobserver segmentation variability, which is known to contribute to QR feature instability.^23^

More recently, 3D-printed imaging phantoms have been used to evaluate QR feature robustness.^24^^–^^26^ In one study,^26^ a realistic liver phantom was constructed by first converting the patient images into surface models using stereolithography (STL) file formats. However, the STL format does not capture the internal structure or texture of images and only represents the shell or surface of the modeled object.^27^ In another study,^24^ simulated lung nodules were 3D printed and inserted into a chest anthropomorphic phantom to evaluate QR feature robustness. Although the approach demonstrates the possibility of voxel-based 3D printing, some of the methods are complex, requiring simulations that may be limited by the extent to which models of anatomy and the imaging system are realistic enough.^28^ Other approaches include using standard desktop inkjet printers with ink cartridges that are filled with aqueous potassium iodide solutions to generate realistic 3D prints.^29^^,^^30^ The doped ink is deposited either on standard or specialized paper. Our proposed method has the distinct advantage of going directly from a digital imaging and communication in medicine (DICOM) CT scan to a 3D printer, using commercially available technology. Consequently, we can overcome key issues, such as the lack of adhesion between layers, coarser resolution, and requirement for extensive simulations that are seen with other methods.^31^

To overcome the limitations, we evaluate the feasibility of translating anatomy seen on a CT scan to a physical phantom using a multimaterial 3D printer with commercially available voxel-printing software (PolyJet Objet 260 Connex 3, Stratasys, Eden Prairie, Minnesota). The proposed method uses voxel printing technology to (i) develop fit-for-use, custom-designed 3D-printed phantom’s, imprinted with actual tumor patterns seen on CT exams, to QC and validate QR feature robustness and (ii) evaluate the repeatability and reproducibility of derived QR features.

## 2 Materials and Methods

### 2.1 Study Participants

Institutional review board approval was obtained, and the requirement for informed consent was waived.

We modeled the 3D-printed radiomic phantom after diseased tissues seen on CT scans of six unique patients. Four patients had pancreatic adenocarcinoma (PDAC), one patient had nonsmall cell carcinoma (NSCLC), and one patient presented with advanced hepatic cirrhosis. We chose these patient scans because of the heterogeneous appearance of the diseased tissues. The patients with PDAC and advanced hepatic cirrhosis received contrast-enhanced abdominal CT exams using a 64 slice CT scanner (Discovery CT750 HD; GE Medical Systems, Milwaukee, Wisconsin) with the following scan parameters: tube voltage of 120 kVp, noise index of 14, tube current modulation ranging from 220 to 380 mA, 0.7 s rotation time, and pitch of 0.984. The images were reconstructed using a 512×512 matrix, the filtered back projection reconstruction algorithm, and a standard convolution kernel. The reconstructed slice thickness was 2.5 mm, with an interval of 2.5 mm. Intravenous contrast administration included 150 mL of iodinated contrast material at 4  mL/s (Iohexol 300  mgI/mL, Omnipaque 300, GE Healthcare, Cork, Ireland), respectively. The tumors were manually outlined by radiologists on the axial scans [window/level: 400/40 Hounsfield unit (HU)] using Volume Viewer on Advantage volume share 7 (GE Medical Systems, Milwaukee, Wisconsin). The CT scan of the patient with NSCLC was from a publicly available dataset hosted by the Cancer Imaging Archive.^20^^,^^32^^,^^33^ The patient was imaged on a 16-slice CT scanner (Lightspeed, General Electric, Madison, Wisconsin). Images were reconstructed using a standard and lung convolution kernel. The scan data from the standard convolution kernel were used in this study. Further details of image acquisition parameters are available in the associated publication.^20^ Across all patient scans, the in-plane pixel size ranged from 0.695 to 0.977 mm (mean=0.851  mm). The tumors’ maximum diameter ranged from 21 to 61 mm, with a mean of 46 mm. The largest tumor diameters were manually measured on a transverse image plane viewed with Volume Viewer on Advantage volume share 7 (GE Medical Systems, Milwaukee, Wisconsin) by an experienced radiologist. The placement of the tumors within the background cirrhotic liver was arbitrary.

### 2.2 Phantom Model Fabrication

**Fig. 1:**
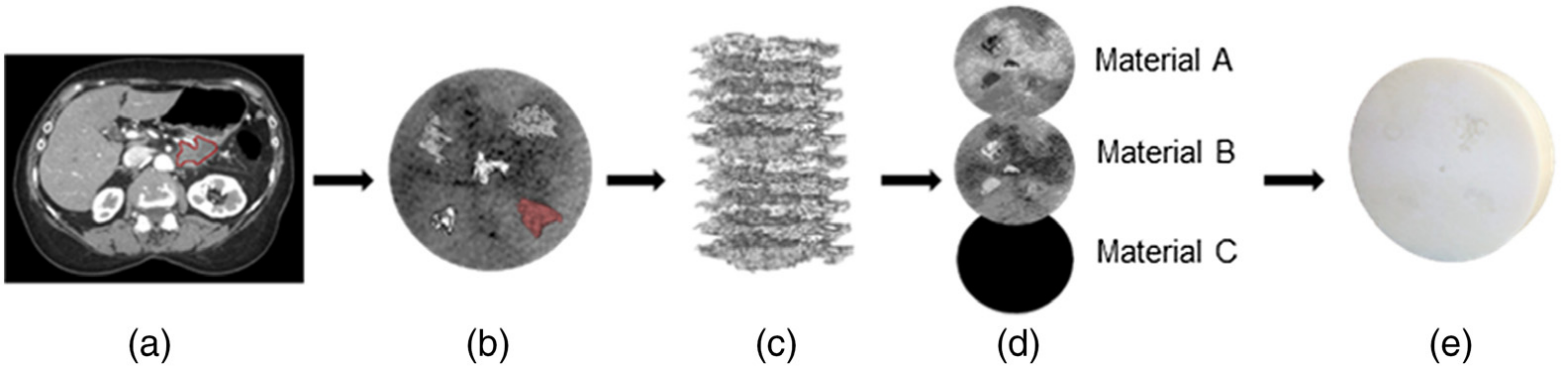
Workflow to generate 3D-printed phantom. (a) The tumor was segmented from the patient CT exam. A cross-sectional slice from a single patient’s CT image shows the contoured PDAC. (b) Binary masks of the tumors were generated and then used to replace the background voxel values with the tumor voxel intensity values. (c) The combined volume was then supersampled to the resolution of the 3D printer and stacked into slices. Each slice from (c) was then dithered using the Floyd–Steinberg dithering algorithm into binary raster files. (d) Three sets of raster files were generated, one for each resin material. These files define the spatial location of each resin material. (e) Resultant 3D print of the combined volume. Due to the material used, visualizing the internal structure is not possible with the naked eye.

Figures 1(a)–1(e) show a graphical overview of the workflow used to 3D print the radiomic phantom. The multimaterial 3D printer used in this study can simultaneously deposit up to three different photopolymer resins.^34^ The resolution of a single droplet of resin in the x-y direction and the layer thickness (the z-direction) is on the order of 48×84×30  μm, which is smaller than the resolution of a typical CT scanner (∼0.5×0.5×0.6  mm).^34^ The selection of printing material was determined by scanning several solid samples of available resin materials using the patient abdominal CT protocol described in Sec. 2.1 and measuring the HU values. The two materials that had the highest and lowest HU values were selected.

At first, an experienced radiologist segmented each tumor and a circular portion of the cirrhotic liver from the scans at their original resolution. The segments were then individually normalized to have voxel intensity values between 0 and 1. The normalized ratios were used to determine the proportion of resin material deposited into a single voxel.^35^ Binary images of the individual tumor volumes were then used to mask an area over the cirrhotic liver images [Fig. 1(b)]. Next, we merged the volumes without modifying the original HU values or the normalized ratios of each scan. The combined slices, as shown in Fig. 1(b), were then supersampled using the Whittaker–Shannon (SINC) interpolation method to the resolution of the 3D printer [Fig. 1(c)]. Finally, each slice was dithered using the Floyd–Steinberg dithering algorithm into binary raster files.^36^ The raster files encode the spatial location over which each material is deposited [Fig. 1(d)]. The disc shape of the phantom was designed to have a diameter of 18 cm and thickness of 4 cm. These dimensions were selected so that the disc would fit within a tissue equivalent enclosure that was originally used with a commercially available low-contrast helical CT QC phantom (model 061, CIRS).

Since the printer can simultaneously print with three different resin materials, three sets of bitmap files were generated, one set for each resin material. Within the first raster file, a value of 1 indicates the deposition of material A, and a value of 0 indicates that material A will not be deposited. The second set of raster files (material B) was generated by inverting material A files so that a value of 0 now had a value of 1. The third set of bitmaps consisted of all zeros since two materials with opposing densities were enough to generate the desired contrast differences. The resulting 3D print is shown in Fig. 1(e).

### 2.3 Computed Tomography Scan Modes

**Fig. 2:**
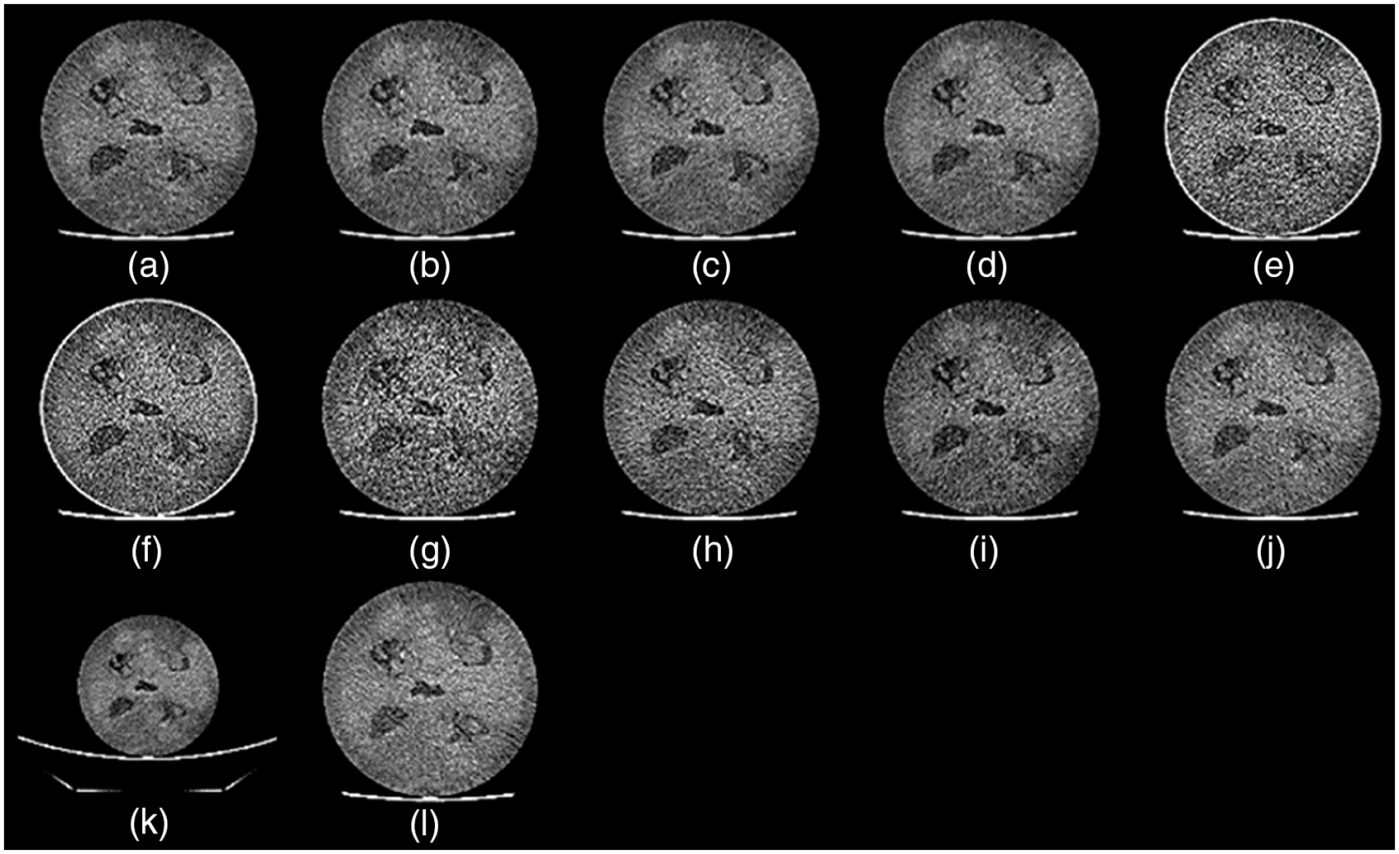
Cross-sectional CT images of the radiomic phantom from each scan mode evaluated in this study. For each image, a window width of 40 and a level of 100 were applied. The differences between each scan mode are (a) baseline image reconstructed with standard kernel. (b) ASiR of 10%. (c) ASiR 20% and (d) ASiR 30%. (e) Lung kernel. (f) Lung kernel with ASiR 40%. (g) Bone kernel. (h) Reduced tube current of 100 mA. (i) Reduced tube potential of 100 kVp. (j) Dual-energy CT image reconstructed at monochromatic 70 keV. (k) Enlarged DFOV of 350 mm with a pixel size of 0.689 mm. (l) Phantom placed off-center by 30 mm. The off-center image was electronically centered within the field of view.

A 64 slice CT scanner (HD750, General Electric, Madison, Wisconsin) was used to acquire 30 repeat scans of the radiomic phantom. The scanning parameters were as follows: 120 kVp, 280 mA, 0.7 s, pitch of 0.984, filtered back projection algorithm with a standard kernel, total collimation of 40 mm, display field of view (DFOV) 250 mm, reconstructed slice thickness, and interval of 1.25 mm. The phantom was centered in the gantry using the system onboard laser alignment lights. The associated volume CT dose index was 15.96 mGy. The average radiomic feature values determined from this protocol were considered the reference in percent deviation (pDV) calculations.^37^ The deviation from the reference was determined by rescanning the phantom, sequentially, five times, without movement between scans using the scan modes listed in Table 1. All parameters of the reference protocol remained fixed while each scan mode was implemented. Figure 2 shows cross-sectional axial slices of the radiomic phantom scanned using each additional scan mode. In addition to commonplace scan modes, such as different tube potentials and currents, we evaluated QR feature robustness with adaptive statistical iterative reconstruction (ASiR), and the phantom positioned vertically off-center by 30 mm in the inferior direction. The latter^38^ is a practice commonly observed in the clinic. We evaluate its impact on QR features in this study because the off-center placement of the patient within the CT gantry misplaces the thickest portion of the bow-tie filter relative to the patient’s anatomy, which leads to increased beam hardening artifacts and, consequently, increased noise or variability of CT HU values.^39^ ASiR is a feature that reduces the pixel noise standard deviation while preserving structural detail and is available in 10 different strengths. As the strength or percentage of ASiR increases, the noise magnitude decreases, noise texture becomes coarser and more uniform, and images generally appear smoother.^40^^,^^41^ The strengths and combinations that we use in this study are based on what we use in our clinic. The DFOV dictates the pixel size in the x-y direction. As DFOV increases, the size of the pixel increases, and the resolution in the x-y direction decreases. The rapid switching dual-energy CT variant used in this study acquires two projections nearly simultaneously while operating at a low and high peak tube potential of 80 and 140 kVp. With the two projections, reconstructing many image types is possible; these include virtual monochromatic images (VMI) that depict anatomy from the viewpoint of a monochromatic x-ray source ranging in energy from 40 to 140 keV or material density images. For this study, we chose to evaluate radiomic feature robustness on DECT scans reconstructed with a VMI of 60 keV. The scan modes were chosen due to their use in the clinic.

**Table 1:** Additional scan modes included in the study. The options were chosen due to their frequent use in the clinic.

Additional scanning modes	
Convolution kernel	Standard, lung and bone kernels^a^
ASiR	ASiR 10%, 20%,^b^ and 30%
Peak tube potential (kVp)	100
Tube current (mA)	100
Phantom off-center in the y-axis inferior direction (mm)	30
DFOV	350 mm (0.684 mm pixel size)
Dual energy CT	Virtual monochromatic energy: 60 keV

a Reference kernel.

b Also performed as a combination of the lung kernel and ASiR 20%.

### 2.4 Radiomic Feature Extraction

The computational environment for radiobiological research (CERR)^42^ was used to extract the prognostic QR features listed in Table 2. We chose these features because previous literature reports illustrated their potential prognostic capabilities for NSCLC and PDAC. They were extracted from the original images without any preprocessing, such as image smoothing or interpolation of voxel sizes, and the settings used for feature calculation were as follows: (1) the images were discretized using a fixed bin width of 25. (2) The average value of each texture feature was computed over all 13 directions to obtain rotational invariance. (3) For the prognostic PDAC QR features, images were discretized using a bin width of 25 and a patchwise volume of 2×2×2  mm voxels. Detailed descriptions of the feature definitions can be found in Refs. 42–44.

**Table 2:** Prognostic radiomic features analyzed for repeatability and deviation relative to reference values.

Radiomic features	
NSCLC^43^	First-order energy^a^
	GLRLM: gray-level non-uniformity (GLN)^b^
	HLH wavelet preprocessed GLRLM GLN^c^
PDAC^2^	First-order energy^a^
	First-order entropy^a^
	GLCM-contrast^d^
	GLCM-dissimilarity^d^

Note: GLRLM, gray-level run length matrix; NSCLC, nonsmall cell lung carcinoma; PDAC, pancreatic ductal adenocarcinoma.

a First-order statistical features.

b Gray-level run length matrix feature.

c Wavelet-based feature.

d Gray-level co-occurrence matrix texture features.

### 2.5 Statistical Analysis

The structural similarity index (SSIM)^45^ was used to calculate the similarity between the original cirrhotic liver and the resulting 3D print. Repeatability (i.e., precision) of radiomic features was evaluated using the within-subject coefficient of variation (wCV, %):^46^

$$\mathrm{wCV}\%=\frac{\sigma_{w}}{\mu }\times 100,$$ (1)

where $\sigma_{w}$ is the within-subject standard deviation and μ is the mean of individual radiomic features. A wCV% of <10% was considered to be repeatable. The 95% confidence interval (CI) was calculated using chi-squared $\left(x^{2}\right)$ as the pivotal statistic as follows:

$$\mathrm{CI}(95\%)=\sqrt{\frac{Nxw({\mathrm{wCV}}^{2})}{x_{n,\propto }^{2}}},$$ (2)

where N is the number of tumors and $x_{n,\propto }^{2}$ is the percentile of the distribution with n degrees of freedom. The lower bound α is 0.975, and the upper bound α is 0.025.

The pDV (%) of radiomic feature derived from the additional scan modes was calculated as follows:

pDV(%)=(fn¯−fo^  fo^±δpDV)×100, (3)

where $\overset{¯}{f_{n}}$ is the average value of the radiomic feature extracted from images of each tumor across the different scanning parameters and fo^ is the average of the reference value, as described above.

The one-sample Wilcoxon signed-rank test was used to determine equality between the reference feature and the median feature value derived from the additional scan modes. A p-value of <0.05 was considered significant. The effect size was calculated as

$$r=\frac{Z}{\sqrt{N_{\mathrm{obs}}}},$$ (4)

where Z is the z-score, $N_{\mathrm{obs}}$ is the number of observations, and r ranges from −1 to 1. The 95th percentile CIs for the effect size estimate were determined using 100 bootstrap samples. All statistical analyses were completed using RStudio.^47^

## 3 Results

### 3.1 Hounsfield Unit of Printing Materials and Structural Similarity

**Fig. 3:**
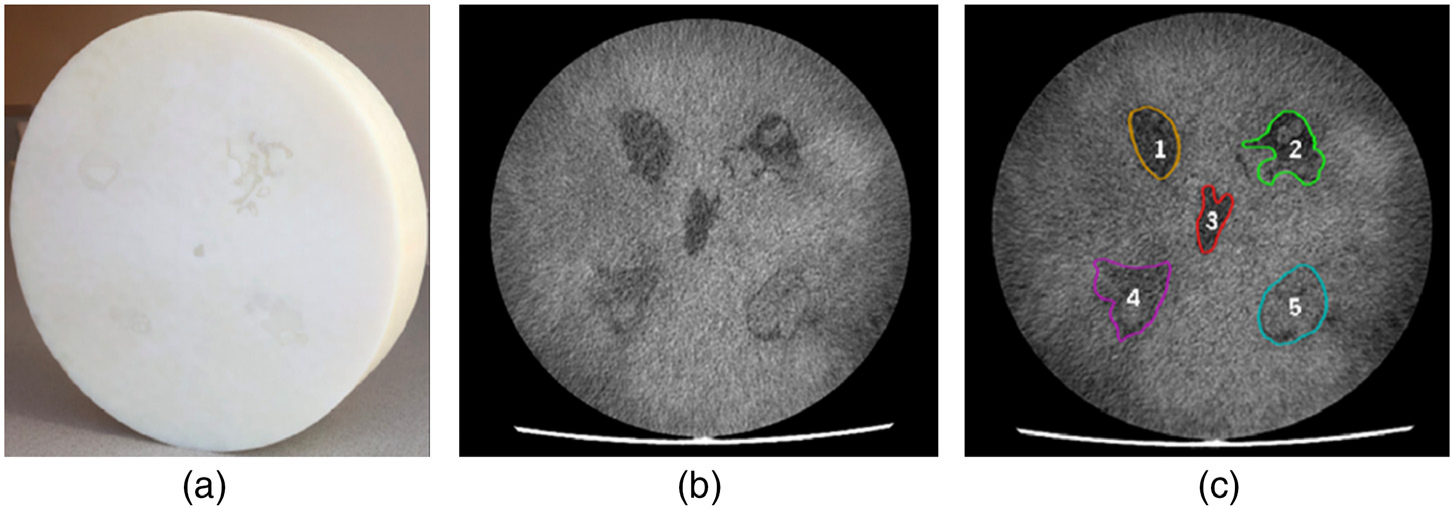
(a) The 3D-printed radiomic phantom. (b) Axial slice generated from a CT scan shows the embedded tumors within the background tissue. (c) The tumor contours were generated, and the tumor types were labeled as 1–Non-small cell lung carcinoma; 2 to 5–Pancreatic ductal adenocarcinoma.

**Fig. 4:**
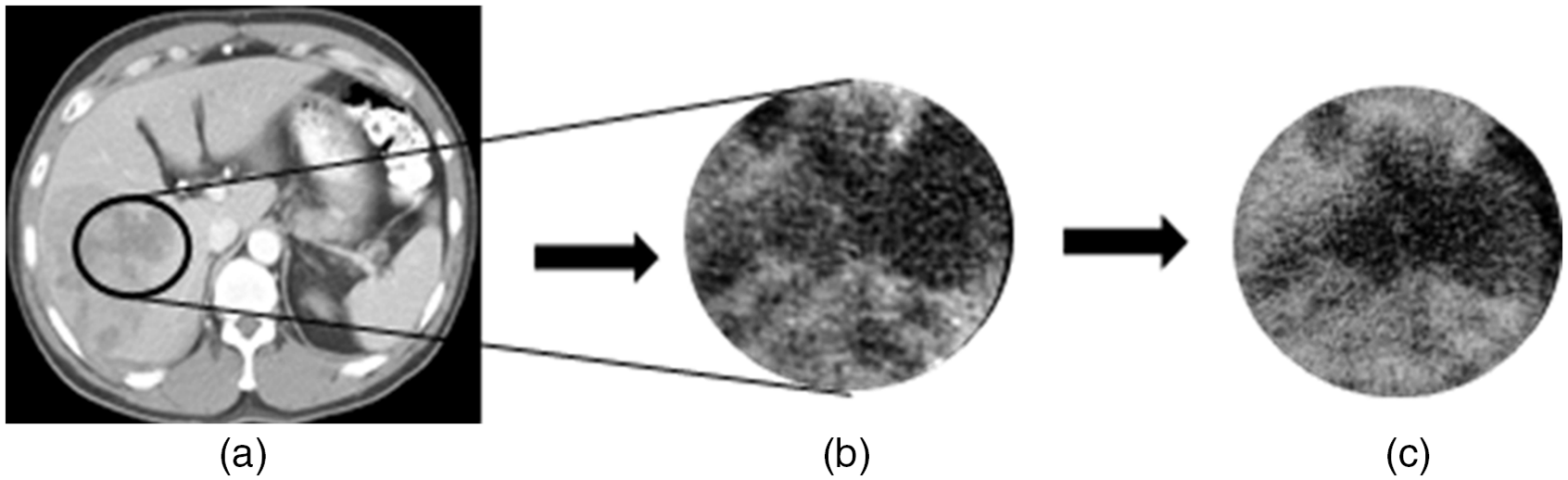
(a) An axial slice from a patient CT showing the region of interest (ROI) around the heterogeneous hepatic tissue. (b) A cropped and expanded view of the portion of the patient’s liver on which the background of the 3D print was modeled. (c) An axial slice of the resulting 3D print CT scan.

Figure 3 shows the resulting 3D-printed phantom, a cross-sectional CT scan, and the physician drawn contours overlaid onto each tumor. The overall time taken to 3D print the phantom was ∼8  h. The final radiomic phantom was circular, with a measured diameter of 176±0.2  mm and an axial length of 42±0.2  mm. The two-resin materials with the lowest (65±5  HU) and highest (125±5  HU) CT numbers were VeroWhite (material A) and TangoPlus (material B). The SSIM between the cirrhotic liver background [Fig. 4(b)] and resultant 3D print [Fig. 4(c)] was 0.71. An SSIM value closer to 1 suggests more similarity between images.

### 3.2 Repeatability and Percent Deviation

**Fig. 5:**
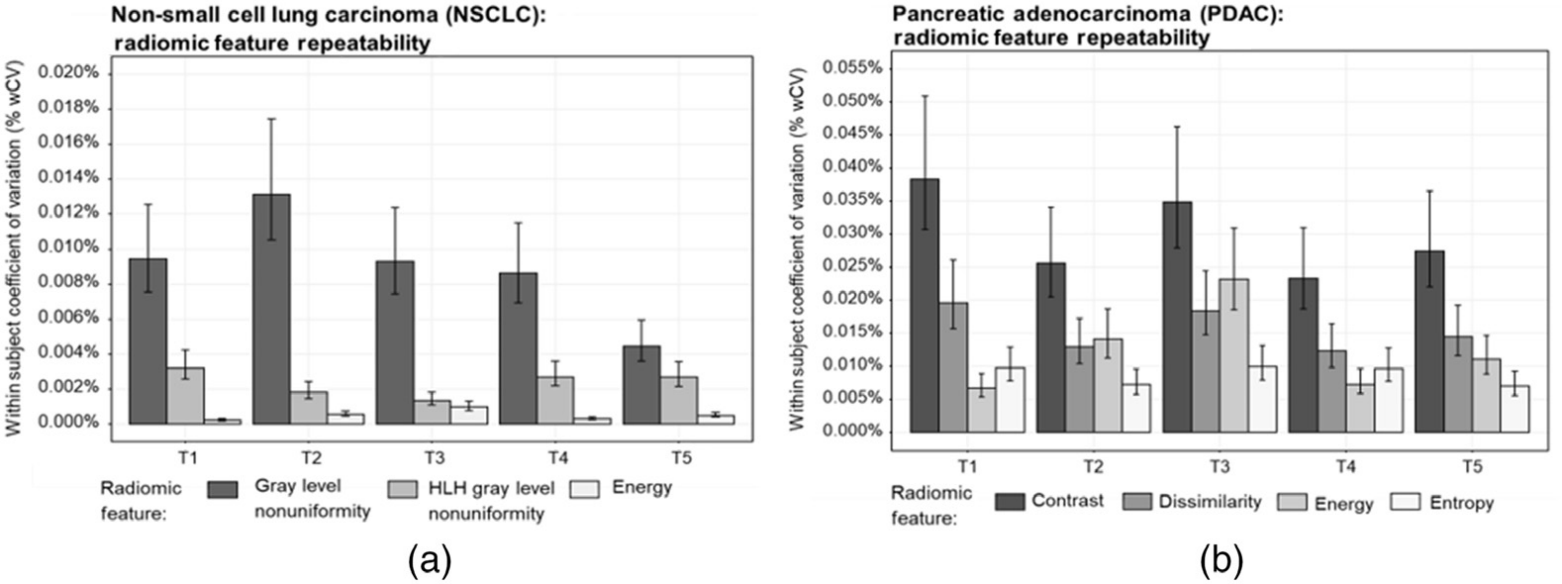
The within-subject coefficient of variation (wCV, %) for prognostic non-small cell lung carcinoma radiomic features extracted from each tumor. The wCV was computed from the 30 repeated CT scans acquired with the reference protocol. The 95th percentile confidence intervals are displayed for each feature value.

**Fig. 6:**
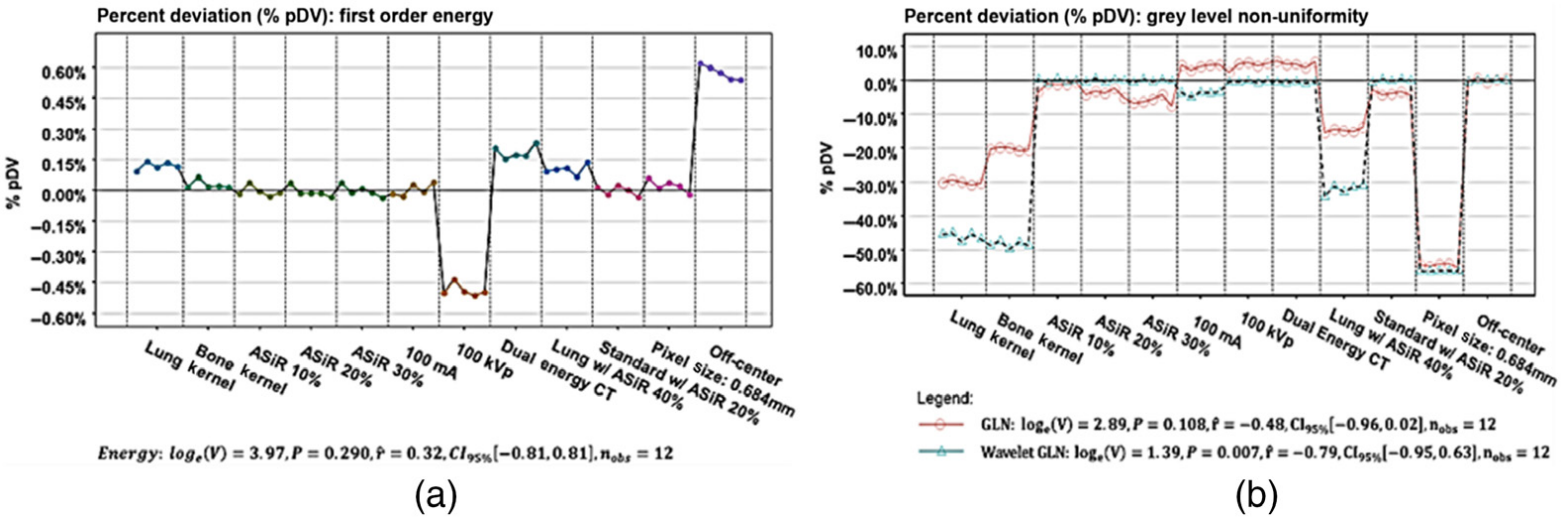
The percent deviation (pDV, %) and one-sample Wilcoxon signed-rank test compare the prognostic NSCLC features. Comparisons are being made between the average feature value derived from the 30 repeat scans and the additional scan modes. (a) First-order energy, (b) gray- level non-uniformity (GLN), and HLH wavelet GLN.

**Fig. 7:**
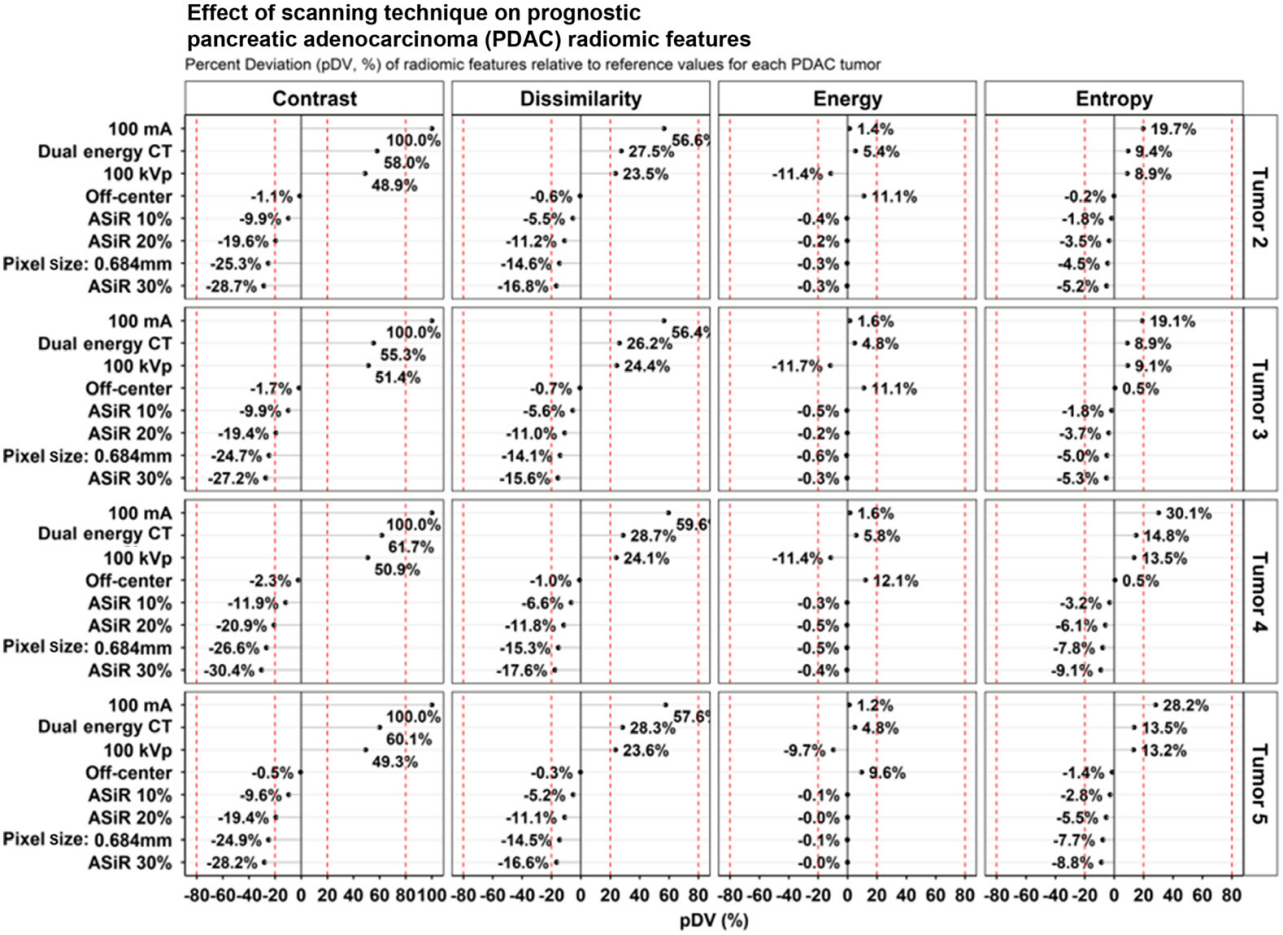
Dot plots of the change in radiomic feature values as a function of each scanning technique and tumor type.

The repeatability of the prognostic NSCLC and PDAC radiomic features is shown in Fig. 5, where wCV (%) <1.0% across features. Figure 6 shows the pDV for the NSCLC radiomic features. The average pDV of first-order energy was 0.01% (range: −0.49% to 0.89%, p=0.290) across all scan modes. The average pDV of GLRLM gray-level non-uniformity (GLN) was 10.2% (range: −55.2% to 5.57%, p=0.108) [Fig. 6(b)]. The application of ASiR 40% to images reconstructed with the lung kernel resulted in the pDV for GLRLM GLN decreasing by a factor of 2, from −30% to −15%. The average pDV for HLH GLN pDV was 15.7% (range: −56.3% to 0.52%, p=0.007). Similar to GLRLM GLN, pDV for HLH GLN decreased when ASiR 40% was applied to images reconstructed with the lung kernel. Figure 7 shows the pDV of prognostic PDAC radiomic features across scan modes. With the application of ASiR 10% to 30%, the pDV for contrast, dissimilarity, and entropy increased in the negative direction for all tumors, but overall, the deviation remained below 30%. Across all tumor types, the pDV for GLCM-contrast and GLCM-dissimilarity exceeded 40% and 20% when the phantom was scanned with the reduced dose scan modes and with the application of DECT. In addition, the pDV for GLCM-contrast was ≤10% when the phantom was scanned with a larger pixel size of 0.689 mm and the different strengths of ASiR.

## 4 Discussion

We have devised a method to 3D print what is seen on a CT scan to a physical imaging phantom using commercially available, multimaterial 3D-printing technology and software Voxel print. The method provides a straightforward approach to producing fit-for-use phantoms that validate longitudinally stable QR feature values in a clinical setting.

The results in this study also illustrate that previously discovered prognostic QR features were repeatable but sensitive to different scan modes. In contrast to previous works in which uniform phantoms were used,^48^ we observe that, across all scan modes, first-order energy was not significantly affected by the reconstruction algorithm or other scan modes (pDV=±1.0%, P>0.290). The deviation for GLN and HLH GLN increased as image noise increased, but the pDV reduced with the application of ASiR 40%. The findings suggest that increased scrutiny or exclusion of GLN and HLH GLN is warranted for future studies. The lack of reproducibility in some QR features, as noted by the high pDV, demonstrates the need for improved validation approaches. A correlation between QR features derived from tumors seen on abdominal CT scans and the underlying tumor microenvironment needs to be interpreted with caution.

The concept of quantifying diseased tissue seen on CT scans is not new. Efforts to quantify bone mineral density (BMD) from CT scans date to the mid-1970s.^49^^,^^50^ However, similar to the issues that plague current QR efforts, BMD measurements were variable across scanning protocols and devices.^50^ To address the lack of standardization and variability across CT scanners, the European spine phantom (ESP) was developed. It was designed to be a practical but effective tool that could standardize and cross calibrate BMD measurements made across CT scans. During its redesign in the 1990s, an international consortium published critical characteristics that a quantitative CT QC phantom should possess.^50^ The characteristics included a phantom that is (1) geometrically defined with realistic dimensions, (2) fit-for-use across CT scanners, (3) closely anthropomorphic so that standard patient protocols can be used without alteration, and (4) composed of limited materials with a range of attenuation characteristics, so linearity can be assessed. Because of technological advances in CT scanning hardware and software, efforts to develop robust QR features would benefit from employing phantoms that meet the design criteria used to manufacture the ESP.

Additional caution is required when interpreting results derived from phantoms data. The applicability or translation of results from phantom studies to the clinic depends on the realism of the phantom components.^51^ The radiomic phantom is crudely anthropomorphic (i.e., it lacks the fat planes or does not incorporate the influence of beam hardening artifacts from contrast or bone). However, the purpose of the phantom is to establish the minimum performance requirement for the QR features^51^ and inform about the continued stability as CT scanning technology evolves. A distinct advantage of the radiomic phantom is that, by incorporating realistic shapes and patterns, it offers tests for additional quantities, such as measuring tumor diameter, volume, or the comparative performance of manual, semiautomated, and automated segmentation techniques. In general, 3D printing offers the opportunity to generate the ground-truth value for parameters to be measured.^24^ Although multimaterial 3D printing offers several advantages relative to previous approaches, some issues requiring further attention can be addressed with additional investigations. First, the density and HU value of available photopolymer resins is limited, but recent investigations into doping agents show that the HU value range of current resins could be increased.^52^ Second, the size of the 3D print bed restricts the maximum anatomical area that could be printed. The Objet 260 printer used in this study can reproduce a volume with a maximum size of 255×252×200  mm. However, the size of phantoms may be overcome by designing modular phantoms. Third, the phantom remained stationary during imaging and was not able to assess the impact of motion.

## 5 Conclusions

The strategy proposed here is to derive ROI tumor and normal tissue features from human CT scans and then to custom print a CT phantom capturing a facsimile of those features. Such a phantom can then be used to measure the variability of imaging features as well as the stability of the overall QR feature, and has the potential to be used as part of the QR QA process. In measurements using our prototype, we found that some previously reported prognostic radiomic features are noisy in practice and need to be used with caution or preferably excluded from clinical signature implementations. Personalized, custom-designed phantoms present a flexible, yet practical, way to validate and compare QR signatures over time and across systems.
